# Resistance to insecticides and synergism by enzyme inhibitors in *Aedes albopictus* from Punjab, Pakistan

**DOI:** 10.1038/s41598-020-78226-0

**Published:** 2020-12-03

**Authors:** Hafiz Azhar Ali Khan

**Affiliations:** Institute of Agricultural Sciences, University of the Punjab, Lahore, Pakistan

**Keywords:** Ecology, Zoology

## Abstract

The widespread use of insecticides has ecological consequences such as emergence of insecticide resistance and environmental pollution*. Aedes albopictus* is a major vector of dengue virus in the Punjab province, Pakistan. Control of *Ae. albopictus* with insecticides along with source eradication is critical in the prevention and control of dengue fever but is threatened by the development of insecticide resistance. Here, field strains of *Ae. albopictus* from eight cities of Punjab were evaluated for resistance against temephos, deltamethrin and permethrin. For temephos, high resistance (RR_LC50_ > tenfold) was found in larvae of the Rawalpindi strain, moderate resistance (RR_LC50_ = five- to tenfold) in Multan, Faisalabad, Sialkot, Lahore and Sheikhupura strains, and low resistance (RR_LC50_ < fivefold) in Kasur and Sahiwal strains. In the case of deltamethrin, high resistance was seen in adults of the strain from Faisalabad, moderate resistance in the strains from Sialkot, Sheikhupura, Lahore and Kasur, and low resistance in Sahiwal, Multan and Rawalpindi strains. For permethrin, adults of all the field strains exhibited high levels of resistance. In synergism bioassays, toxicity of all the insecticides in the field strains significantly enhanced when tested in combination with piperonyl butoxide or *S,S,S*-tributylphosphorotrithioate, suggesting the probability of metabolic-based mechanisms of resistance. In conclusion, field strains of *Ae. albopictus* from Punjab exhibit resistance to temephos, deltamethrin and permethrin, which might be associated with metabolic mechanisms of resistance.

## Introduction

Arboviruses, viruses transmitted by mosquitoes or other arthropods, cause severe diseases and deaths in humans worldwide. These are transmitted by multiple species of insects and each species has its own importance depending upon their geographical distribution, virus carrying capacity, and their potential of virus transmission^1^. *Aedes albopictus* (Skuse) is an anthropophilic mosquito species with the potential to act as a competent vector of important arboviruses such as dengue, chikungunya, yellow fever, Rift Valley fever and Zika. *Aedes albopictus* has worldwide distribution^2^, which makes it an important factor for the transmission of arboviral-diseases in different parts of the world.

Of different arboviral-diseases, dengue fever has been considered as one of the most rapidly spreading diseases in the world with an estimate of 3.9 billion people in different countries at risk^3^. This disease has also affected different regions of Pakistan. In Pakistan, the first case of dengue fever was reported in 1994, and country’s worst epidemics of dengue fever were observed between 2011–2013^4,5^. Lack of vaccination is one of the major hurdles in the management of dengue fever. Although, a vaccine (Dengvaxia) to prevent dengue fever has been registered in several countries, it is not yet in widespread use and has certain safety concerns^1,6^. *Aedes albopictus* is one of the major vectors of dengue virus in Pakistan having wide distribution in different parts of the country. In the absence of vaccines and effective drugs for dengue fever, control of *Ae. albopictus* with insecticides coupled with breeding-source eradication is therefore absolutely critical in the prevention and control of dengue fever.

In Pakistan, control of *Ae. albopictus* is principally based on chemical measures coupled with community participation for larval source reduction. Although different alternative measures for *Aedes* mosquitoes such as the use of genetic manipulation, sterile insect techniques, and the use of endosymbionts such as *Wolbachia* that compete with dengue virus and hinders its reproduction have the potential to give satisfactory results, they are recommended/practiced in restricted locations worldwide. Since the development and adoption of new control measures are time taking processes, hence the current insecticide-based management strategies would be expected to play an important role in *Aedes* mosquitoes management for many years to come^1^. Currently, temephos (an organophosphate) through larviciding, and deltamethrin and permethrin (pyrethroids) through space and indoor residual sprays have been in wide use in *Aedes* mosquitoes control programs. In this scenario, wide use of insecticides is fraught with the rapid development of insecticide resistance and environmental pollution^7,8^.

Insecticidal bioassays in combination of enzyme inhibitors (e.g., *S,S,S*-tributylphosphorotrithioate (DEF or tribufos) and piperonyl butoxide (PBO)) to check their synergistic effect on insecticide toxicity is a rapid and inexpensive approach to provide clues regarding the possibility metabolic mechanisms of insecticide resistance^9,10^. Studies revealed that esterase- and/or oxidase-based mechanism of resistance could be present in insect species if toxicity of a particular insecticide is enhanced when used in combination with DEF, since DEF inhibits the activities of oxidases and esterases. Likewise, possibility of oxidase-based mechanism of resistance could be detected if the toxicity of an insecticide is synergized when used in combination with PBO, since it suppresses activities of oxidases^9,11,12^. Recently, development of insecticide resistance to organophosphate (temephos) and pyrethroid (deltamethrin and permethrin) insecticides have been reported in another important vector of dengue virus, *Ae. aegypti* (L.), from different areas of Punjab^5^,and bioassays in combination of PBO and DEF suggested the probability of metabolic mechanism of resistance associated with resistance to the organophosphate. Likewise, there are chances of resistance development in *Ae. albopictus* that may reduce the efficacy of current insecticide-based control programs. Therefore, present study was planned to investigate the possibility of insecticide resistance development to commonly used insecticides (temephos, deltamethrin and permethrin) in *Ae. albopictus* collected from the urban areas of Punjab, Pakistan. Furthermore, synergism bioassays by using PBO and DEF in combination with insecticides were also performed to find the clues about the presence of metabolic mechanisms of resistance in the putatively resistant strains of *Ae. albopictus*.

## Materials and methods

### *Aedes albopictus* strains

The study was conducted on *Ae. albopictus* strains collected from eight cities of the Punjab province, Pakistan, during 2017–2018 (Table 1). The cities were selected on the basis of dengue fever outbreaks in recent years and wide use of insecticides application for control of *Aedes* mosquitoes. A reference strain (Ref-S) of *Ae. albopictus* was also collected from an area of very low chemical application and reared in the laboratory for 20 generations in insecticide-free environment. Although not truly susceptible, the susceptibility of the Ref-S strain to insecticides was quite higher as compared to the field strains (see results section) and hence can be used as baseline for resistance monitoring^13^. Immature *Ae. albopictus* were collected from different breeding sites and transported to the laboratory. About 500–700 immature *Ae. albopictus* were used to start each colony. In laboratory these strains were reared by maintaining 26 ± 1 °C, 65 ± 5% r.h. and photoperiod of 12:12 (L:D) h. In the laboratory, the diet was consisted of fish food for larvae and 20% sucrose solution for adult mosquitoes, while female *Ae. albopictus* were bloodfed from an anesthetized mouse thrice a week following in accordance with relevant guidelines and regulations. The field strains were reared up to F1 or F2 generations before starting bioassays. The study/bioassay protocols used against *Ae. albopictus* were performed according to the standard guidelines and regulations, and approved by the bioethics committee of Institute of Agricultural Sciences, University of the Punjab, Lahore.

**Table 1:** Collection history of *Aedes albopictus* field strains used for resistance monitoring and synergism experiments.

Collection locality	Collection period	Collection site	Coordinate
Multan	September, 2017	Water cooler, tires	30.1575° N, 71.5249° E
Sahiwal	September, 2017	Discarded small containers, flower pots, water air cooler	30.6682° N, 73.1114° E
Lahore	July, 2018	Flower pots, tree holes, irrigation channel	31.5204° N, 74.3587° E
Sheikhupura	July, 2017	Tree holes, water air cooler	31.7167° N, 73.9850° E
Kasur	September, 2018	Tree holes, flower pots	31.1179° N, 74.4408° E
Faisalabad	August, 2018	Tree holes, tires, water air cooler	31.4504° N, 73.1350° E
Sialkot	September, 2017	Tree holes, irrigation channel, water air cooler	32.4945° N, 74.5229° E
Rawalpindi	August, 2018	Tree holes, flower pots	33.5651° N, 73.0169° E

### Insecticides and synergists

Bioassays were conducted by using technical-grade temephos (> 95%), deltamethrin (99.5%), permethrin (98%), PBO (98%), and DEF (98%) (ChemService Inc., West Chester, PA).

### Bioassays

Bioassay methods have been described in detail elsewhere^5^. Briefly, toxicity of temephos was checked through larval bioassay in three replicates. A range of dilutions were prepared in acetone, while acetone alone was taken as a control. For Ref-S and field strains, the concentrations used were ranged between 0.01 to 0.32 µg/ml and 0.04 to 1.28 µg/ml, respectively.

Bottle-bioassay method developed by the Centre for Disease Control (CDC) was followed to check the toxicity of deltamethrin and permethrin against female adults of *Ae. albopictus*^9^. Deltamethrin and permethrin were dissolved in acetone to prepare concentrations. These concentrations were used to coat 250 ml glass bottles at the rate of 1 ml/bottle. Control bottles were coated with acetone alone. Range of concentrations used were as follows: 0.31 to 10 µg/bottle for deltamethrin against the Ref-S strain; 1 to 32 µg/bottle for deltamethrin against field strains; 0.125 to 4 µg/bottle for permethrin against the Ref-S strain; 1 to 32 µg/bottle for permethrin against field strains. In each coated bottle 25 unfed females (3 to 5 days old) were introduced and the knockdown effect recorded after 1 h. After this period, females were shifted in insecticide free flasks. Additional details are provided in supplemental materials.

### Synergism experiments

Synergism bioassays were performed as outlined in our previous report^5^. In brief, *Ae. albopictus* larvae (for temephos) or unfed females (for deltamethrin or permethrin) were exposed to PBO and DEF solutions for 1 h. After exposure to synergists, larvae or adults were then exposed to different concentrations (*n* = 25 per concentration per replicate) of insecticides via the insecticide solution (for larvae against temephos) or insecticide-treated bottles (for adults against deltamethrin or permethrin) as stated in the bioassay section. Additional details are provided in supplemental materials.

### Data analyses

Data of the knockdown effect and mortality were subjected to Probit analysis using the software PoloPlus^14^. Median knockdown concentrations (KC_50s_) for deltamethrin and permethrin were calculated from the data after 1 h exposure. Median lethal concentrations (LC_50s_) for temephos, deltamethrin and permethrin were determined from the mortality data after 24 h exposure. KC_50s_ or LC_50s_ values of field strains were divided with those of the Ref-S strain to get resistance ratios (RR_KC50_, RR_LC50_). The ratios were classified according to the following scale: RR < 5 folds (low resistance); RR ranged from 5 to 10 folds (moderate resistance); RR > 10 folds (high resistance)^5,15^.

Simple linear regression was performed to find the association between LC_50_ and KC_50_ values for deltamethrin or permethrin in eight field strains. The resultant slope vales were analyzed following the criterion of Flores, et al.^16^: a slope value ≃ 1 indicates LC_50_ ≃ KC_50_ and most of the knockdown mosquitoes are dead after 24 h exposure; a slope value > 1 indicates the LC_50_ value is greater than the KC_50_ value most of the knockdown mosquitoes recovered after 24 h exposure and more insecticide concentration is needed to cause mortality of these mosquitoes^16^.

## Results

The results of bottle-bioassays using adults of *Ae. albopictus* for estimating KC_50_ and corresponding RR values for deltamethrin and permethrin are presented in Table 2. The results revealed the highest susceptibility of the Ref-S strain to deltamethrin and permethrin with KC_50_ values 1.42 and 0.60 µg/ml, respectively. KC_50_ values of different field strains ranged from 4.35 to 18.55 µg/ml for deltamethrin, and 6.85 to 15.47 µg/ml for permethrin. Field strains showed significant levels of RR values at KC_50_ level when compared with the Ref-S strain, based on the 95% CIs of RR values did not include 1. In the case of deltamethrin, Multan, Rawalpindi and Sahiwal strains exhibited low resistance (RR 3.06, 3.39 and 3.50 fold, respectively), Sialkot, Kasur, Faisalabad and Lahore moderate resistance (RR 5.02, 6.02, 8.94 and 9.80 fold, respectively), and high resistance in the strain of Sheikhupura (13.06 fold) (Table 2).

**Table 2:** Knock-down concentrations (KC_50s_) and resistance ratios (RR_KC50_) of adults of *Aedes albopictus* strains against deltamethrin and permethrin.

Insecticide	Strain	n	KC_50_ (95% CI) (µg/ml)	Fit of probit line				RR_KC50_ (95% CI)
				Slope (S.E)	χ^2^	df	*p*^£^	
Deltamethrin	Ref-S	525	1.42 (0.94–2.25)	2.33 (0.20)	7.83	4	0.10	1
	Multan	525	4.35 (3.27–6.25)	1.92 (0.17)	5.47	4	0.24	3.06 (2.40–3.90)*
	Sahiwal	525	4.97 (3.70–7.35)	1.83 (0.17)	5.15	4	0.27	3.50 (2.70–4.51)*
	Lahore	450	13.91 (9.58–27.98)	2.06 (0.24)	3.96	3	0.27	9.80 (7.29–13.12)*
	Sheikhupura	525	18.55 (15.36–23.35)	1.96 (0.19)	1.81	4	0.77	13.06 (10.07–16.88)*
	Kasur	525	8.55 (6.54–11.40)	1.85 (0.16)	4.67	4	0.32	6.02 (4.75–7.60)*
	Faisalabad	525	12.69 (10.31–16.18)	1.53 (0.15)	1.21	4	0.88	8.94 (6.80–11.70)*
	Sialkot	525	7.13 (5.75–9.22)	1.54 (0.16)	3.14	4	0.54	5.02 (3.79–6.63)*
	Rawalpindi	450	4.82 (4.13–5.64)	2.23 (0.20)	1.64	3	0.65	3.39 (2.72–4.21)*
Permethrin	Ref-S	525	0.60 (0.40–0.90)	2.41 (0.22)	6.69	4	0.15	1
	Multan	450	7.28 (6.06–8.81)	1.82 (0.19)	2.61	3	0.46	12.13 (9.56–15.45)*
	Sahiwal	525	7.96 (6.64–9.66)	1.68 (0.14)	2.49	4	0.65	13.27 (10.44–16.92)*
	Lahore	450	6.85 (5.75–8.34)	1.99 (0.21)	0.70	3	0.87	11.42 (9.00–14.53)*
	Sheikhupura	525	8.50 (6.81–11.21)	1.55 (0.15)	1.27	4	0.87	14.19 (10.62–18.97)*
	Kasur	525	12.24 (9.69–16.20)	1.32 (0.13)	1.40	4	0.84	20.40 (15.19–27.50)*
	Faisalabad	525	8.69 (6.98–11.10)	1.33 (0.13)	1.69	4	0.79	14.48 (11.00–19.13)*
	Sialkot	525	15.47 (11.99–21.38)	1.28 (0.14)	2.59	4	0.63	25.78 (18.69–35.69)*
	Rawalpindi	525	12.99 (10.58–16.54)	1.56 (0.15)	1.07	4	0.90	21.65 (16.57–28.40)*

RR_KC50_, resistance ratio at KC_50_ = (KC_50_ of a field strain) ÷ (KC_50_ of Ref-S).

*significantly different from Ref-S based on 95% CIs of RR_KC50_ did not include 1^39^.

Table 3 displays LC_50_ values and corresponding RR values obtained for temephos (against larvae), deltamethrin and permethrin (against adults) in different strains of *Ae. albopictus*. All the insecticides showed the highest toxicity to the Ref-S strain with LC_50_ values 0.05, 1.48 and 0.54 µg/ml for temephos, deltamethrin and permethrin, respectively. The LC_50_ values for different field strains ranged from 0.17 to 0.64 µg/ml for temephos, 4.20 to 28.84 µg/ml for deltamethrin, and 6.44 to 37.14 µg/ml for permethrin. High resistance to temephos was found in the Rawalpindi strain (RR 12.80 fold), moderate resistance in Multan (RR 6.00 fold), Faisalabad (RR 6.00 fold), Sialkot (RR 7.80 fold), Lahore (RR 8.00 fold) and Sheikhupura (RR 8.20 fold) strains, and low resistance in Kasur (RR 3.20 fold) and Sahiwal (RR 3.40 fold) strains. In the case of deltamethrin, high resistance was seen in the strain of Faisalabad (RR 19.50 fold), moderate resistance in the strains of Sialkot (RR 6.44 fold), Sheikhupura (RR 7.51 fold), Lahore (RR 7.58 fold) and Kasur (RR 7.82 fold), and low resistance in Sahiwal (RR 2.84 fold), Multan (RR 3.51 fold) and Rawalpindi (RR 4.97 fold) strains. For permethrin, all the field strains exhibited high resistance with RR values ranged from 11.93 to 68.78 fold (Table 3).

**Table 3:** Lethal concentrations (LC_50s_) and resistance ratios (RR_LC50_) of *Aedes albopictus* larvae against temephos, and adults against deltamethrin and permethrin.

Insecticide	Strain	n	LC_50_ (95% CI) (µg/ml)	Fit of probit line				RR_LC50_ (95% CI)*
				Slope (S.E)	χ^2^	df	*p*^£^	
Temephos	Ref-S	525	0.05 (0.04–0.07)	2.88 (0.24)	4.79	4	0.31	1
	Multan	525	0.30 (0.26–0.35)	2.26 (0.20)	2.84	4	0.58	6.00 (4.47–6.79)
	Sahiwal	600	0.17 (0.13–0.22)	2.59 (0.19)	8.46	5	0.13	3.40 (2.57–3.79)
	Lahore	450	0.40 (0.34–0.46)	2.28 (0.20)	1.04	3	0.79	8.00 (5.92–8.90)
	Sheikhupura	525	0.41 (0.35–0.48)	2.26 (0.21)	1.37	4	0.85	8.20 (6.03–9.13)
	Kasur	525	0.16 (0.14–0.19)	1.97 (0.16)	1.06	4	0.90	3.20 (2.38–3.64)
	Faisalabad	525	0.30 (0.23–0.38)	2.68 (0.24)	5.72	4	0.22	6.00 (4.51–6.65)
	Sialkot	525	0.39 (0.32–0.49)	1.97 (0.23)	0.36	4	0.99	7.80 (5.52–9.17)
	Rawalpindi	525	0.64(0.51–0.85)	1.48 (0.15)	2.85	4	0.58	12.80 (9.59–17.03)
Deltamethrin	Ref-S	525	1.48 (1.13–1.92)	2.88 (0.22)	7.89	4	0.10	1
	Multan	450	4.66 (3.99–5.56)	2.31 (0.22)	2.35	3	0.50	3.15 (2.55–3.89)
	Sahiwal	450	4.20 (3.51–5.15)	1.88 (0.19)	2.65	3	0.45	2.84 (2.25–3.58)
	Lahore	450	11.22 (8.08–18.90)	1.96 (0.22)	3.27	3	0.35	7.58 (5.82–9.89)
	Sheikhupura	525	11.11 (8.70–14.69)	2.06 (0.17)	4.62	4	0.33	7.51 (6.07–9.30)
	Kasur	525	11.56 (9.50–14.47)	1.60 (0.15)	0.31	4	0.99	7.82 (6.10–10.01)
	Faisalabad	525	28.84 (21.52–43.54)	1.44 (0.17)	0.52	4	0.97	19.50 (13.49–28.19)
	Sialkot	525	9.52 (7.63–12.59)	1.63 (0.17)	0.67	4	0.95	6.44 (4.86–8.52)
	Rawalpindi	450	7.36 (5.92–9.60)	1.52 (0.18)	1.82	3	0.61	4.97 (3.79–6.53)
Permethrin	Ref-S	525	0.54 (0.43–0.68)	2.31 (0.18)	4.80	4	0.31	1
	Multan	450	11.82 (8.58–18.16)	2.39 (0.23)	5.10	3	0.16	21.89 (17.57–27.36)
	Sahiwal	525	8.71 (6.54–12.07)	1.81 (0.15)	5.51	4	0.24	16.13 (12.80–20.41)
	Lahore	450	8.83 (7.35–11.05)	1.98 (0.21)	2.18	3	0.54	16.35 (12.74–21.05)
	Sheikhupura	525	6.44 (4.28–11.53)	1.48 (0.14)	7.72	4	0.10	11.93 (9.06–15.77)
	Kasur	525	16.19 (12.00–24.20)	1.80 (0.17)	5.10	4	0.28	29.98 (23.19–38.38)
	Faisalabad	525	18.08 (14.10–23.48)	1.39 (0.15)	2.71	4	0.61	33.48 (24.39–46.10)
	Sialkot	525	37.14 (25.74–64.56)	1.25 (0.16)	2.57	4	0.63	68.78 (43.10–110.06)
	Rawalpindi	525	19.50 (14.80–24.16)	1.27 (0.14)	2.18	4	0.70	36.11 (25.49–51.31)

RR_LC50_, resistance ratio at LC_50_ = (LC_50_ of a field strain) ÷ (LC_50_ of Ref-S).

*Significantly different from Ref-S based on 95% CIs of RR_LC50_ did not include 1^39^.

**Figure 1:**
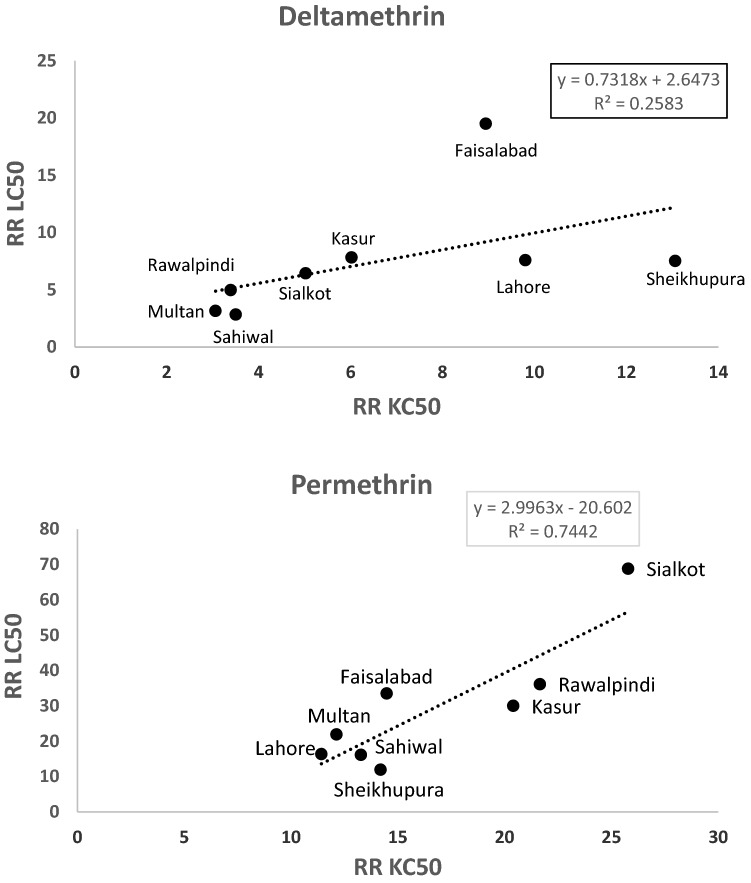
Simple linear regression between LC50 and KC50 values of adult *Ae. albopictus* of different field strains against deltamethrin or permethrin.

Regression analysis between RR_LC50_ and RR_KC50_ for deltamethrin and permethrin is shown in Fig. 1. For deltamethrin, the slope value 0.73 indicates that majority of the knockdown mosquitoes failed to recover after 24 h. In contrast, the slope value 2.99 for permethrin revealed that most of the mosquitoes recovered after knockdown and a higher concentration of permethrin is required to eventually kill mosquitoes (Fig. 1).

The results of synergism bioassays against Ref-S and selected field strains are shown in Table 4. The data revealed that the toxicity of all the insecticides in the Ref-S strain did not change significantly when bioassayed in the presence of either PBO or DEF. The synergism ratios were non-significant based on 95% CIs of SRs include 1. In the case of field strains, toxicity of all the insecticides enhanced significantly when bioassayed in the presence of PBO or DEF (based on synergism ratio test and non-overlapping 95% CIs of LC50 values), suggesting the possibility of metabolic mechanism of resistance. Toxicity of temephos against larvae of *Ae. albopictus* enhanced 2.25 and 2.66 fold in the presence of DEF and PBO, respectively. Similarly, toxicity of deltamethrin enhanced by 2.64 fold (+ DEF) and 2.07 fold (+ PBO), while toxicity of permethrin increased by 1.94 fold (+ DEF) and 2.62 fold (+ PBO) against adults of *Ae. albopictus* (Table 4).

**Table 4:** Toxicity of temephos, deltamethrin, and permethrin with or without synergist in *Aedes albopictus* strains.

Strain	Treatment	LC_50_ (95% CI) (µg/ml)	Fit of probit line				SR£(95% CI)
			Slope (S.E)	χ^2^	df	*p*^£^	
Ref-S	Temephos	0.05 (0.04–0.07)	2.88 (0.24)	4.79	4	0.31	
Ref-S	Temephos + DEF	0.05 (0.03–0.06)^ns^	2.42 (0.18)	4.24	4	0.37	1.00 (0.89–1.32)
Ref-S	Temephos + PBO	0.06 (0.04–0.07) ^ns^	2.69 (0.20)	6.94	4	0.14	0.84 (0.76–1.11)
Ref-S	Deltamethrin	1.48 (1.13–1.92)	2.88 (0.22)	7.89	4	0.10	–
Ref-S	Deltamethrin + DEF	1.25 (0.92–1.70) ^ns^	2.51 (0.19)	9.05	4	0.06	1.18 (0.97–1.43)
Ref-S	Deltamethrin + PBO	1.31 (0.98–1.73) ^ns^	2.13 (0.17)	6.43	4	0.17	1.13 (0.92–1.38)
Ref-S	Permethrin	0.54 (0.43–0.68)	2.31 (0.18)	4.80	4	0.31	–
Ref-S	Permethrin + DEF	0.56 (0.42–0.76) ^ns^	2.27 (0.17)	7.65	4	0.11	0.96 (0.77–1.18)
Ref-S	Permethrin + PBO	0.49 (0.36–0.67) ^ns^	2.49 (0.19)	8.85	4	0.06	1.10 (0.89–1.34)
Rawalpindi	Temephos	0.64 (0.51–0.85)	1.48 (0.15)	2.85	4	0.58	
Rawalpindi	Temephos + DEF	0.29 (0.23–0.38)*	1.35 (0.14)	2.60	4	0.63	2.25 (1.56–3.25)**
Rawalpindi	Temephos + PBO	0.24 (0.19–0.32)*	1.38 (0.14)	2.31	4	0.68	2.66 (1.87–3.78)**
Faisalabad	Deltamethrin	28.84 (21.52–43.54)	1.44 (0.17)	0.52	4	0.97	–
Faisalabad	Deltamethrin + DEF	10.94 (8.26–15.98)*	1.31 (0.14)	0.92	4	0.92	2.64 (1.64–4.24)**
Faisalabad	Deltamethrin + PBO	13.92 (10.46–19.22)*	1.45 (0.17)	2.08	4	0.72	2.07 (1.28–3.35)**
Sialkot	Permethrin	37.14 (25.74–64.56)	1.25 (0.16)	2.57	4	0.63	
Sialkot	Permethrin + DEF	19.17 (14.65–24.35)*	1.30 (0.15)	1.12	4	0.89	1.94 (1.13–3.33)**
Sialkot	Permethrin + PBO	14.18 (10.53–20.87)*	1.60 (0.15)	4.39	4	0.36	2.62 (1.59–4.31)**

£SR, synergism ratio = (LC_50_ of temephos, deltamethrin or permethrin alone) ÷ (LC_50_ of temephos, deltamethrin or permethrin plus PBO or DEF).

ns, non-significant (*p* > 0.05) based on overlapping 95% CI of LC_50_ values of insecticides plus PBO or DEF when compared with that of the LC_50_ of insecticide alone.

**Significant SR, 95% CIs of SR did not include 1^39^.

## Discussion

Control failure of insect pests due to insecticide resistance often results in high dosage of insecticides, which ultimately pollute the environment^17,18^. The present study provides an evidence of resistance development to temephos, deltamethrin and permethrin in *Ae. albopictus* collected from different cities of the province Punjab. The selected cities have reported cases of dengue fever every year since the country’s major epidemic in 2011. For this reason, the use of insecticides has been most frequent to control dengue mosquitoes in order to combat dengue fever epidemics, which might be the leading cause of resistance development in *Ae. albopictus*. Insecticides from different classes are heavily used in Punjab for the management of different insect pests, including *Ae. albopictus*. As a consequence, a number of studies have reported development of insecticide resistance in different disease vectors from Pakistan such as *Musca domestica* L.^19,20^ *Ae. aegypti*^5,21,22^, *Culex quinquefasciatus* Say^23^, *Anopheles* spp.^24,25^, and *Periplaneta americana* L.^26^.

Previously, we have reported resistance development in *Ae. albopictus* from cropping areas of Punjab as a consequence of indirect exposure to different agrochemicals^27^; however, there are limited reports of resistance development in *Ae. albopictus* from urban areas. For instance, Arslan, et al.^22^ reported the probability of resistance to deltamethrin and permethrin in *Ae. albopictus* from Rawalpindi. Similarly, Mohsin, et al.^21^ reported probability of resistance to different insecticides in *Ae. albopictus* from the Lahore city of Punjab. However, the scope of both studies, in our opinion, was limited since mosquitoes were sampled from only one location in each study. Therefore, it was the need to explore other important areas of the province with dengue positive cases every year. In our study, we examined insecticide resistance in more detail by determining resistance ratios in *Ae. albopictus* from eight major cities with respect to dengue fever incidence, and also studied the possibility of metabolic mechanism of resistance.

Pyrethroids such as deltamethrin and permethrin are dominant insecticides for the management of urban insect pests in residential environments. The results of the present study revealed low to high levels of resistance to deltamethrin and high levels of resistance to permethrin in different field strains of *Ae. albopictus*. In addition, regression analysis was performed to find the association between LC_50_ and KC_50_ values for deltamethrin or permethrin in different field strains. For deltamethrin, the slope value indicated that majority of the knockdown mosquitoes failed to recover after 24 h. In contrast, the slope value for permethrin revealed that most of the mosquitoes recovered after knockdown and a higher concentration of permethrin was required to eventually kill mosquitoes. These insecticides are widely used in Punjab to combat mosquitoes. The most probable reason for resistance *Ae. albopictus* could be the fact of usage of these insecticides in different forms such as fogging, indoor residual sprays, mosquito coils against *Aedes* mosquitoes in order to protect from dengue fever^5^. Furthermore, injudicious use of pyrethroid insecticides for different insect pests in urban settings^19^ could also be responsible for resistance development in different strains of *Ae. albopictus*. Recently, we have reported resistance to permethrin and deltamethrin in *Ae. aegypti* from 12 cities (Faisalabad, Gujranwala, Lahore, Multan, Okara, Pattoki, Rawalpindi, Sahiwal, Sargodha, Sheikhupura, Sialkot and Kasur) of Punjab, Pakistan^5^. Except the Okara strain, all field strains exhibited high levels of resistance to permethrin. In case of deltamethrin, low levels of resistance were found in Multan, Okara and Sahiwal strains, moderate levels of resistance in Sialkot, Gujranwala and Sargodha strains, and high levels of resistance in Kasur, Pattoki, Lahore, Sheikhupura, Faisalabad and Rawalpindi. Previously, resistance to pyrethroid in *Aedes* mosquitoes has also been reported worldwide^28–32^.

In the present study, low to moderate levels of resistance to temephos were found in seven field strains, while the Rawalpindi strain exhibited high resistance to temephos. In Punjab, temephos is the most widely used as larvicide to manage larvae of *Aedes* mosquitoes that might be responsible for resistance development to temephos in *Ae. albopictus*. Temephos resistance in *Ae. aegypti* has also been reported from different cities (stated above) of Punjab^5^. Of these, high levels of resistance were found in Gujranwala, Lahore, Kasur, Rawalpindi and Faisalabad strains, moderate levels of resistance in Okara, Sahiwal, Pattoki, Sialkot and Sheikhupura, and low levels of resistance in Sargodha and Multan. There are variable reports of resistance development to temephos in *Ae. albopictus* from different areas of the world. For instance, Ponlawat, et al.^32^ reported low levels of resistance to temephos in *Ae. albopictus* from different localities of Thailand. Larvae of *Ae. albopictus* from Selangor, Malaysia were found to be highly resistant to temephos^33^. In contrast, larvae of *Ae. albopictus* from Central Africa were found to be susceptible to temephos^34^. The different expression of resistance might be due to differences in geographic origin of *Ae. albopictus* strains, insecticide exposure histories and/or different environmental conditions.

Insecticidal bioassays in combination of enzyme inhibitors (e.g., PBO or DEF) to check their synergistic effect on insecticide toxicity is a rapid and inexpensive approach to provide clues regarding the possibility metabolic mechanisms of insecticide resistance^9,10^. Studies revealed that esterase- and/or oxidase-based mechanism of resistance could be present in insect species if toxicity of a particular insecticide is enhanced when used in combination with DEF, since DEF inhibits the activities of oxidases and esterases. Likewise, possibility of oxidase-based mechanism of resistance could be detected if the toxicity of an insecticide is synergized when used in combination with PBO, since it suppresses activities of oxidases^9,11,12^. In the present study, toxicity of temephos, deltamethrin and permethrin have been evaluated in combination with PBO or DEF against Ref-S and selected field strains of *Ae. albopictus* to check the possibility of metabolic mechanisms of resistance. The results showed that there was no synergistic effect of all the insecticides when checked against Ref-S. strain. It was expected, since Ref-S strain has been maintained in insecticide free environment. However, there was a significant synergistic effect of PBO and DEF on the toxicity of all the insecticides in field strains of *Ae. albopictus*. These results indicate the possibility of esterase- and oxidase-based mechanisms of resistance linked with insecticide resistance in field strains. Both of these enzyme inhibitors have also been reported to enhance toxicity of temephos in *Ae. aegypti* from Cucuta^35^ and in different field strains of *Ae. aegypti* from Punjab, Pakistan^5^.

Resistance to pyrethroid insecticides in insect pests can be due to the enhanced activities of metabolic enzymes and/or modification to the target site of these insecticides. Sayyed, et al.^12^ reported the synergistic effect of PBO and DEF on the toxicity of deltamethrin in *Chrysoperla carnea* (Stephens). In contrast to the present study, toxicity of deltamethrin and permethrin did not synergize by enzyme inhibitors in field strains of *Ae. aegypti*^5^.In the present study, the Sialkot strain showed about 69 fold RR at LC_50_ level, and its toxicity was enhanced by < threefold in the presence of PBO or DEF. This high level of resistance to permethrin indicates the possibility of altered target site mechanism responsible for resistance to permethrin. Smith, et al.^36^ reported metabolic detoxification and altered target site as the major mechanisms of resistance to pyrethroid insecticides in *Ae. albopictus* and *Ae. aegypti*. However, altered target site mechanism alone was found to be the major mechanism of resistance to pyrethroid in *Ae. aegypti* in Puerto Rico^37^. Multiple mechanisms could be present at the same time in resistant mosquitoes^38^, depending upon the history and geographical origin of insect strains. Future studies may be planned at molecular level to further confirm the mechanisms of resistance in *Ae. albopictus*.

Insecticide resistance is a major hindrance in the management of *Aedes* mosquitoes that ultimately lead to seasonal outbreaks of dengue fever in different areas of Pakistan. An important strategy could be to lessen the use of insecticides by adopting integrated vector management (IVM) tool such as mosquito-breeding source reduction, the use of mosquito nets, and management of rainwater collecting bodies. *Aedes albopictus* from different cities of Punjab, Pakistan, have shown resistance development to insecticides used for mosquito control, which may result in severe outbreaks of dengue fever in the future. To avoid this situation, regular insecticide resistance monitoring along with the use of alternative measures could be effective tools for managing *Ae. albopictus*.

## Supplementary information

Supplementary Information.
