# Supplementary material for: Resistance to insecticides and synergism by enzyme inhibitors in Aedes albopictus from Punjab, Pakistan
Source: Sci Rep. 2020 Dec 3;10:21034. doi: 10.1038/s41598-020-78226-0 (PMC7713067; doi:10.1038/s41598-020-78226-0)
Supplement: Supplementary file 1 — Supplementary Information. [file 41598_2020_78226_MOESM1_ESM.docx]

**Resistance to temephos, deltamethrin and permethrin, and synergism by enzyme inhibitors in *Aedes albopictus* from Punjab, Pakistan**

Hafiz Azhar Ali Khan

Institute of Agricultural Sciences, University of the Punjab, Lahore.

Correspondence at: azhar.iags@pu.edu.pk

Bioassays*

Toxicity of temephos was checked against *Ae. albopictus* larvae by the methodology proposed by the World Health Organization [^1^](#_ENREF_1). For this purpose, five to six concentrations of temephos were prepared causing mortality between 2 and 98%. These concentrations were prepared in acetone while acetone alone was taken as a control. For reference and laboratory strains, the concentrations used were in the range of 0.01 to 0.32 µg/ml and 0.04 to 1.28 µg/ml, respectively. Three replications were performed and 25 late 3^rd^ instar larvae or early 4^th^ instar larvae were used against each concentration and control. Mortality data was recorded 24 h after exposure and larvae were considered dead when they show no movement when touched with a needle [^2^](#_ENREF_2)

For deltamethrin and permethrin, the CDC bottle bioassay protocol formulated by the Centre for Disease Control and Prevention was used against adults of *Ae. albopictus* [^3^](#_ENREF_3). Different concentrations of deltamethrin and permethrin causing mortality between 2 and 98% were prepared in acetone and used to coat 250 ml glass bottles at the rate of 1 ml/bottle. Control bottles were coated with acetone alone. Range of concentrations used were as follows: 0.31 to 10 µg/bottle for deltamethrin against the Ref-S strain; 1 to 32 µg/bottle for deltamethrin against field strains; 0.125 to 4 µg/bottle for permethrin against the Ref-S strain; 1 to 32 µg/bottle for permethrin against field strains. In each coated bottle 25 unfed females (3 to 5 days old) were introduced and the knockdown effect recorded after 1 hour. After this period, females were shifted in an insecticide free flask having a cotton boll soaked in 20% sugar–water solution. Final mortality was recorded 24 h after the 1-h insecticide exposure [^2^](#_ENREF_2).

Synergism bioassays were performed as outlined in our previous report [^2^](#_ENREF_2). Briefly, PBO and DEF solutions were prepared in acetone, and glass bottles were coated with 1 ml of either PBO (400 μg/bottle) or DEF (125 μg/bottle) solution in three replicates as per the protocol proposed by the CDC for synergism studies [^3^](#_ENREF_3). Control bottles were coated with acetone alone. In case of deltamethrin and permethrin, female mosquitoes were exposed to the synergist-coated bottles including control for 1 h, and afterward exposed to different concentrations (*n*=25 per concentration per replicate) of insecticides via the insecticide-treated bottles as stated in the bioassay section. For temephos, PBO and DEF solutions (400 and 125 μg/ml, respectively) were prepared in acetone. Late 3^rd^ instar or early 4^th^ instar larvae were exposed to the synergist solution of PBO or DEF for 1 h, and then exposed to different concentrations of temephos as stated in the bioassay section. Synergism bioassays were replicated three times.

*Adapted from our previous report [^2^](#_ENREF_2)

References:

1 WHO. Instructions for determining the susceptibility or resistance of mosquito larvae to insecticides. (World Health Organization, 1981).

2 Khan, H. A. A. & Akram, W. Resistance Status to Deltamethrin, Permethrin, and Temephos Along With Preliminary Resistance Mechanism in Aedes aegypti (Diptera: Culicidae) From Punjab, Pakistan. *J. Med. Entomol.* **56**, 1304-1311 (2019).

3 Brogdon, W. & Chan, A. Guideline for evaluating insecticide resistance in vectors using the CDC bottle bioassay. *USA: CDC Atlanta* (2010).
